# Supplementary material for: Inhibitive Mechanism of Loquat Flower Isolate on Tyrosinase Activity and Melanin Synthesis in Mouse Melanoma B16 Cells
Source: Biomolecules. 2024 Jul 24;14(8):895. doi: 10.3390/biom14080895 (PMC11352878; doi:10.3390/biom14080895)
Supplement: Supplementary file 1 [file biomolecules-14-00895-s001.zip › biomolecules-3007758-supplementary.pdf]

Supplementary Table 1 Full spectrum identification results on constituents of LFP by HPLC-MS/MS

Name	Formula	CAS_num	Class	Annot. DeltaMass [ppm]	Annotation MW	Calc. MW	RT [min]	Area	Relative concentration（μg/g）	Relative percentage（%）
(2S,3S)-2-(3,4-dihydroxyphenyl)-3,7-dihydroxy-2,3-dihydrochromen-4-one	C15 H12 O6	17654-28-3	Flavonoids	-1.4	288.06339	288.06299	6.157	59039386.32	13.4133197	0.026
(9Z,12E)-15,16-dihydroxyoctadeca-9,12-dienoic acid	C18 H32 O4	NA	Fatty Acyls	-1.84	312.23006	312.22948	11.161	960942856.1	218.3192365	0.419
(E)-4-[(1R,2S,3S,4R,8aS)-2,3,4-trihydroxy-2,5,5,8a-tetramethyl-3,4,4a,6,7,8-hexahydro-1H-naphthalen-1-yl]but-3-en-2-one	C18 H30 O4	NA	Prenol lipids	-1.92	310.21441	310.21381	10.912	365044986.6	82.93556923	0.159
1-palmitoyl-2-hydroxy-sn-glycero-3-phosphoethanolamine	C21 H44 N O7 P	53862-35-4	Glycerophospholipids	-1.2	453.28554	453.285	12.679	187639321	42.6302907	0.082
2-(3,4-dihydroxyphenyl)-5,7-dihydroxy-6,8-dimethoxychromen-4-one	C17 H14 O8	NA	Flavonoids	-1.68	346.06887	346.06828	9.953	163340238.2	37.1097156	0.071
3,4,5-trimethoxycinnamic acid	C12 H14 O5	NA	Cinnamic acids and derivatives	-2.22	238.08412	238.0836	9.403	201006116.9	45.66712963	0.088

Name	Formula	CAS_num	Class	Annot. DeltaMass [ppm]	Annotation MW	Calc. MW	RT [min]	Area	Relative concentration （μg/g）	Relative percentage （%）
3-[(2S,3R,4S,5S,6R)-4,5-dihydroxy-6-(hydroxymethyl)-3-[(2S,3R,4S,5S,6R)-3,4,5-trihydroxy-6-(hydroxymethyl)oxan-2-yl]oxyoxan-2-yl]oxy-2-(3,4-dihydroxyphenyl)-5-hydroxy-7-methoxychromen-4-one	C28 H32 O17	259234-17-8	Flavonoids	-1.08	640.16395	640.16326	6.226	199890879.3	45.41375575	0.087
3-Hydroxyphenylacetic acid	C8 H8 O3	621-37-4	Phenols	-0.87	152.04734	152.04721	10.034	68405552.78	15.54124469	0.030
4,4`-Methylen-bis(2-methyl aniline)	C15 H18 N2	838-88-0	Benzene and substituted derivatives	-1.19	226.147	226.14673	7.869	88099499.01	20.01556621	0.038
5,8-dihydroxy-2-(4-hydroxyphenyl)-7-methoxy-3-[(2S,3R,4R,5R,6S)-3,4,5-trihydroxy-6-methyloxan-2-yl]oxychromen-4-one	C22 H22 O11	NA	Others	-1.62	462.11621	462.11546	7.643	452340324.1	102.7684358	0.197
5-(5-methoxycarbonyl-5,8a-dimethyl-2-methylidene-3,4,4a,6,7,8-hexahydro-1H-naphthalen-1-yl)-3-methylpentanoic acid	C21 H34 O4	NA	Prenol lipids	-2.25	350.24571	350.24492	9.414	103437758.8	23.50030742	0.045

Name	Formula	CAS_num	Class	Annot. DeltaMass [ppm]	Annotation MW	Calc. MW	RT [min]	Area	Relative concentration （μg/g）	Relative percentage （%）
7-hydroxy-2-(4-hydroxyphenyl)-5-[(2S,3R,4S,5S,6R)-3,4,5-trihydroxy-6-(hydroxymethyl)oxan-2-yl]oxy-2,3-dihydrochromen-4-one	C21 H22 O10	NA	Others	-1.34	434.1213	434.12071	6.553	219882766.2	49.95576723	0.096
albocycline	C18 H28 O4	25129-91-3	Macrolides and analogues	-0.97	308.19876	308.19846	11.105	77178034.13	17.53428873	0.034
Coumaric acid (isomer of 189, 194)	C9 H8 O3	614-60-8	Cinnamic acids and derivatives	-0.75	164.04734	164.04722	10.271	125316500.7	28.47099865	0.055
Dicaffeoyl Coumaroyl Spermidine	C34 H37 N3 O8	NA	Cinnamic acids and derivatives	-1.42	615.25806	615.25719	8.513	80146077.69	18.20860666	0.035
Dicoumaroyl Spermidine	C25 H31 N3 O4	65715-79-9	Cinnamic acids and derivatives	-1.74	437.23146	437.2307	9.225	732351599.3	166.3849635	0.319
Dihydrokaempferol	C15 H12 O6	480-20-6	Flavonoids	-1.93	288.06339	288.06283	5.946	93906664.93	21.33491212	0.041
Hydroquinone	C6 H6 O2	123-31-9	Phenols	1.02	110.03678	110.03689	9.585	49763831.51	11.30598103	0.022
Isorhamnetin 3-galactoside	C22 H22 O12	6743-92-6	Flavonoids	-1.49	478.11113	478.11041	7.106	4618743000	1049.344859	2.013
Loliolide	C11 H16 O3	5989-02-6	Benzofurans	-0.76	196.10994	196.1098	7.247	236719963.8	53.78105622	0.103

Name	Formula	CAS_num	Class	Annot. DeltaMass [ppm]	Annotation MW	Calc. MW	RT [min]	Area	Relative concentration （μg/g）	Relative percentage （%）
LPC 18:1	C26 H52 N O7 P	19420-56-5	Glycerophospholipids	-1.04	521.34814	521.3476	13.287	59102718.46	13.42770831	0.026
LPC 18:2	C26 H50 N O7 P	NA	Glycerophospholipids	-1.16	519.33249	519.33189	12.442	1105438653	251.1476319	0.482
LPC 18:3	C26 H48 N O7 P	NA	Glycerophospholipids	-1.11	517.31684	517.31626	11.858	413037490.6	93.83911752	0.180
LPE 18:2	C23 H44 N O7 P	85046-18-0	Glycerophospholipids	-1.09	477.28554	477.28502	12.275	276985867.5	62.92917704	0.121
NCGC00380702-01_C20H22O6_2-Butenoic acid, 2-methyl-, (3aR,4R,5R,9aS,9bR)-2,3,3a,4,5,7,9a,9b-octahydro-4-hydroxy-6,9-dimethyl-3-methylene-2,7-dioxoazuleno[4,5-b]furan-5-yl ester, (2Z)-	C20 H22 O6	NA	Fatty Acyls	-1.92	358.14164	358.14095	8.049	133852929.8	30.41041333	0.058
NCGC00380712-01_C19H18O6_1,3-Cyclobutanedicarboxylic acid, 2,4-bis(4-hydroxyphenyl)-, monomethyl ester	C19 H18 O6	NA	Phenols	-1.52	342.11034	342.10982	8.465	235626801	53.53269757	0.103

Name	Formula	CAS_num	Class	Annot. DeltaMass [ppm]	Annotation MW	Calc. MW	RT [min]	Area	Relative concentration （μg/g）	Relative percentage （%）
NCGC00384731-01!(1S,3R,4R,5R)-1,3,4-trihydroxy-5-[(E)-3-(4-hydroxyphenyl)prop-2-enoyl]oxycyclohexane-1-carboxylic acid	C16 H18 O8	1899-30-5	Organooxygen compounds	-1.68	338.10017	338.0996	5.881	336718659.8	76.50003355	0.147
NCGC00385243-01_C12H18O4_{(1R,2R)-2-[(2Z)-5-Hydroxy-2-penten-1-yl]-3-oxocyclopentyl}acetic acid	C12 H18 O4	140631-27-2	Fatty Acyls	-1.72	226.12051	226.12012	5.717	62149782.18	14.11997905	0.027
NCGC00386020-01_C18H28O3_8-{(1S,5R)-4-Oxo-5-[(2Z)-2-penten-1-yl]-2-cyclopenten-1-yl}octanoic acid	C18 H28 O3	67204-66-4	Fatty Acyls	-2.09	292.20384	292.20323	9.938	58654474.81	13.3258706	0.026
PC(16:0/0:0)	C24 H50 N O7 P	17364-16-8	Glycerophospholipids	-1.37	495.33249	495.33181	12.934	833836439.9	189.4415821	0.363
Phenylalanylisoleucine (isomer of 1329)	C15 H22 N2 O3	22951-94-6	Carboxylic acids and derivatives	-1.49	278.16304	278.16263	6.283	77744462.88	17.66297722	0.034
Phloretin + C-Hex, C-Hex	C27 H34 O15	NA	Linear 1,3-diarylpropanoids	-0.98	598.18977	598.18918	6.585	56478282.59	12.83145554	0.025
Phthalic anhydride	C8 H4 O3	85-44-9	Benzofurans	-0.03	148.01604	148.01604	0.046	66986604.01	15.21886983	0.029

Name	Formula	CAS_num	Class	Annot. DeltaMass [ppm]	Annotation MW	Calc. MW	RT [min]	Area	Relative concentration （μg/g）	Relative percentage （%）
Prespatane	C15 H24	NA	Prenol lipids	-1.73	204.1878	204.18745	9.405	173797397.3	39.48550618	0.076
Quercetin-3,4'-O-di-beta-glucoside	C27 H30 O17	29125-80-2	Flavonoids	-1.29	626.1483	626.14749	6.033	1450310365	329.4999797	0.632
Quercitrin	C21 H20 O11	522-12-3	Flavonoids	-1.32	448.10056	448.09997	7.42	385411183.8	87.56262129	0.168
Syringetin-3-O-glucoside	C23 H24 O13	NA	Flavonoids	-1.32	508.12169	508.12102	7.059	328192983.1	74.56306173	0.143
Tricoumaroyl spermidine (isomer of 2952)	C34 H37 N3 O6	NA	Cinnamic acids and derivatives	-1.5	583.26824	583.26736	9.236	6494722487	1475.553771	2.831
(+/-)12(13)-DiHOME	C18 H34 O4	263399-35-5	Fatty Acyls	-1.57	314.24571	314.24522	11.464	120545821.4	27.38713496	0.053
(-)-Caryophyllene oxide	C15 H24 O	1139-30-6	Prenol lipids	-1.13	220.18272	220.18247	8.101	95513848.21	21.70005249	0.042
(-)-Epicatechin	C15 H14 O6	490-46-0	Flavonoids	-1.72	290.07904	290.07854	5.878	4081592405	927.3081453	1.779
(2E,2'E)-N,N'-1,4-Butanediylbis[3-(4-hydroxyphenyl)acrylamide]	C22 H24 N2 O4	NA	Cinnamic acids and derivatives	-1.61	380.17361	380.173	8	492086209.4	111.7984122	0.214
(3R,5R)-1,3,5-Trihydroxy-4-{[(2E)-3-(4-hydroxy-3-methoxyphenyl)-2-	C17 H20 O9	NA	Others	-1.46	368.11073	368.11019	6.133	1035943078	235.3587421	0.452

Name	Formula	CAS_num	Class	Annot. DeltaMass [ppm]	Annotation MW	Calc. MW	RT [min]	Area	Relative concentration（μg/g）	Relative percentage（%）
propenoyl]oxy}cyclohexanecarboxylic acid										
(3β,5ξ,9ξ)-3,23-Dihydroxy-1-oxoolean-12-en-28-oic acid	C30 H46 O5	NA	Prenol lipids	-0.89	486.33452	486.33409	12.724	68481310.82	15.55845636	0.030
(3β,5ξ,9ξ)-3,6,19-Trihydroxyurs-12-en-28-oic acid	C30 H48 O5	NA	Prenol lipids	-1.28	488.35017	488.34955	11.737	201219215.4	45.71554406	0.088
1-Ethyl 6,7-dimethyl 2-oxo-8-oxabicyclo[3.2.1]oct-6-ene-1,6,7-tricarboxylate	C14 H16 O8	NA	Carboxylic acids and derivatives	-1.9	312.08452	312.08393	7.944	1114329156	253.1674896	0.486
1-Linoleoyl glycerol	C21 H38 O4	2277-28-3	Fatty Acyls	-1.64	354.27701	354.27643	15.88	69179318.42	15.71703862	0.030
1-Methyladenine	C6 H7 N5	1670-69-5	Imidazopyrimidines	-0.09	149.07015	149.07013	1.084	55556334.06	12.62199553	0.024
10,16-Dihydroxyhexadecanoic acid	C16 H32 O4	3233-90-7	Fatty Acyls	-1.87	288.23006	288.22952	10.161	1211150744	275.1646511	0.528
13(S)-HOTrE	C18 H30 O3	87984-82-5	Fatty Acyls	-2.02	294.21949	294.2189	9.912	654537592.5	148.7061864	0.285
2'-Deoxyadenosine	C10 H13 N5 O3	958-09-8	Purine nucleosides	-1.46	251.10184	251.10147	1.286	61062775.39	13.873019	0.027

Name	Formula	CAS_num	Class	Annot. DeltaMass [ppm]	Annotation MW	Calc. MW	RT [min]	Area	Relative concentration （μg/g）	Relative percentage （%）
2'-O-Methyladenosine	C11 H15 N5 O4	2140-79-6	Purine nucleosides	-1.38	281.1124	281.11202	1.749	612270876.8	139.1034956	0.267
2,6-Anhydro-1-deoxy-5-O-(6-deoxy-alpha-L-mannopyranosyl) -6-C-[5,7-dihydroxy-2- (4-hydroxyphenyl) -4-oxo-4H-1-benzopyran-6-yl] -xylo-3-hexulose	C27 H28 O13	74158-04-6	Flavonoids	-1.44	560.15299	560.15218	9.502	4990626080	1133.833993	2.175
2,6-di-tert-butyl-4-ethylphenol	C16 H26 O	4130-42-1	Benzene and substituted derivatives	-1.39	234.19837	234.19804	10.16	712152878.6	161.7959609	0.310
2,7-Bis-(4-methyl-piperazine-1-sulfonyl)-4,5-dinitro-fluoren-9-one	C23 H26 N6 O9 S2	NA	Fluorenes	1.66	594.12027	594.12125	7.364	2226213931	505.7796337	0.970
2-(3,4-Dihydroxyphenyl)-5,7-dihydroxy-4-oxo-4H-chromen-3-yl 6-O-β-D-xylopyranosyl-β-D-glucopyranoside	C26 H28 O16	NA	Flavonoids	-1.12	596.13773	596.13707	6.256	51023845.16	11.59224698	0.022
2-Amino-1,3,4-octadecanetriol	C18 H39 N O3	554-62-1	Organonitrogen compounds	-1.35	317.29299	317.29256	10.537	135028230.7	30.67743316	0.059

Name	Formula	CAS_num	Class	Annot. DeltaMass [ppm]	Annotation MW	Calc. MW	RT [min]	Area	Relative concentration （μg/g）	Relative percentage （%）
2-Hydroxycinnamic acid	C9 H8 O3	614-60-8	Cinnamic acids and derivatives	-0.75	164.04734	164.04722	5.88	285094748.2	64.77145583	0.124
3,4,5-Trimethoxyphenyl 6-O-pentopyranosyl-β-D-glucopyranoside	C20 H30 O13	NA	Organooxygen compounds	-1.39	478.16864	478.16798	5.407	53440846.38	12.1413721	0.023
3,4-Dihydroxybenzaldehyde	C7 H6 O3	139-85-5	Organooxygen compounds	-0.77	138.03169	138.03159	5.88	275120542.2	62.50538868	0.120
4'-Methoxyacetophenone	C9 H10 O2	100-06-1	Organooxygen compounds	-0.36	150.06808	150.06803	5.524	75318171.96	17.11174155	0.033
4,6,2',4'-Tetramethoxychalcone 2'-beta-glucoside	C25 H30 O11	NA	Flavonoids	-1.42	506.17881	506.17809	7.94	831405683.3	188.8893318	0.362
4-Anisic acid	C8 H8 O3	100-09-4	Benzene and substituted derivatives	-0.76	152.04734	152.04723	6.127	261852775.6	59.49104849	0.114
4-Indolecarbaldehyde	C9 H7 N O	1047-86-6	Indoles and derivatives	-0.72	145.05276	145.05266	7.464	104894165.9	23.83119253	0.046
4-Methoxybenzaldehyde	C8 H8 O2	123-11-5	Benzene and substituted derivatives	-0.17	136.05243	136.05241	5.952	844718212.3	191.9138417	0.368

Name	Formula	CAS_num	Class	Annot. DeltaMass [ppm]	Annotation MW	Calc. MW	RT [min]	Area	Relative concentration （μg/g）	Relative percentage （%）
4-Methoxycinnamaldehyde	C10 H10 O2	24680-50-0	Cinnamaldehydes	-0.6	162.06808	162.06798	10.091	131201554.2	29.80804005	0.057
4-Methoxycinnamic acid	C10 H10 O3	830-09-1	Cinnamic acids and derivatives	-0.68	178.06299	178.06287	7.181	96681740.95	21.96538924	0.042
4-Pyridineacetic acid	C7 H7 N O2	28356-58-3	Pyridines and derivatives	-0.39	137.04768	137.04762	4.63	110288584.9	25.05676534	0.048
5'-S-Methyl-5'-thioadenosine	C11 H15 N5 O3 S	2457-80-9	5'-deoxyribonucleosides	-1.76	297.08956	297.08904	4.526	379821561.7	86.29269976	0.166
